# Vector species-specific association between natural *Wolbachia* infections and avian malaria in black fly populations

**DOI:** 10.1038/s41598-018-22550-z

**Published:** 2018-03-08

**Authors:** Luke Woodford, Giovanni Bianco, Yoana Ivanova, Maeve Dale, Kathryn Elmer, Fiona Rae, Stephen D. Larcombe, Barbara Helm, Heather M. Ferguson, Francesco Baldini

**Affiliations:** 1 Institute of Biodiversity, Animal Health and Comparative Medicine, University of Glasgow, Glasgow, United Kingdom; 2 Department of Biomolecular Sciences, School of Biology, University of St Andrews, St Andrews, United Kingdom

## Abstract

Artificial infection of mosquitoes with the endosymbiont bacteria *Wolbachia* can interfere with malaria parasite development. Therefore, the release of *Wolbachia*-infected mosquitoes has been proposed as a malaria control strategy. However, *Wolbachia* effects on vector competence are only partly understood, as indicated by inconsistent effects on malaria infection reported under laboratory conditions. Studies of naturally-occurring *Wolbachia* infections in wild vector populations could be useful to identify the ecological and evolutionary conditions under which these endosymbionts can block malaria transmission. Here we demonstrate the occurrence of natural *Wolbachia* infections in three species of black fly (genus *Simulium*), which is a main vector of the avian malaria parasite *Leucocytozoon*. Prevalence of *Leucocytozoon* was high (25%), but the nature and magnitude of its association with *Wolbachia* differed between black fly species. *Wolbachia* infection was positively associated with avian malaria infection in *S. cryophilum*, negatively associated in *S. aureum*, and unrelated in *S. vernum*. These differences suggest that *Wolbachia* interacts with the parasite in a vector host species-specific manner. This provides a useful model system for further study of how *Wolbachia* influences vector competence. Such knowledge, including the possibility of undesirable positive association, is required to guide endosymbiont based control methods.

## Introduction

*Wolbachia* is the most common intracellular bacterium in insects and other invertebrates, infecting an estimated 40% of insect species^1^. As *Wolbachia* is maternally transmitted, infections are often associated with host reproductive manipulation. This reproductive parasitism can prioritise *Wolbachia’s* own reproduction to favour female progeny^2^. The most common reproductive manipulation caused by *Wolbachia* is cytoplasmic incompatibility (CI), which results in embryonic death of any offspring produced from mating between a *Wolbachia*-negative female and a *Wolbachia*-positive male^3–5^. This phenotype allows *Wolbachia* to spread into naive insect populations, as observed in *Drosophila simulans* populations in California between the 1980s and 2000s^6,7^. Another common *Wolbachia*-associated phenotype is pathogen interference. This was first described in *Drosophila melanogaster*, where *Wolbachia* reduces the mortality induced by Drosophila C virus (DCV)^8^, and generally protects against RNA viruses^9^. In the mosquito *Aedes aegypti*, adults infected as embryos with a *Wolbachia* strain isolated from *D. melanogaster* transmitted this endosymbiont to their progeny, a process referred to as stable transinfection. In these transinfected mosquitoes, *Wolbachia* blocked dengue (DENV) and Chikungunya (CHIKV) virus replication^10^. Given *Wolbachia*’s ability to spread into insect populations through CI, the endosymbiont has been successfully introduced into natural *Ae. aegypti* populations to control arbovirus transmission^11^.

Additionally, *Wolbachia* has recently been proposed as a biocontrol agent for malaria^12^, and a number of laboratory-based studies have investigated this possibility. Similar to its impact on RNA viruses, *Wolbachia* has been reported to reduce susceptibility to malaria infection and parasite loads in some instances (e.g. the avian malaria parasite *Plasmodium gallinaceum* in *Ae. aegypti*)^10^. However the degree of infection blocking against malaria is lower than that reported for viruses^13^. Recently, the ability of *Wolbachia* to block *Plasmodium* infection in human malaria vectors (*Anopheles* species) has been tested in the laboratory, but results so far are mixed. A *Wolbachia* strain isolated from *Ae. albopictus* and stably transinfected in *An. stephensi* mosquitoes partly reduces *P. falciparum* development, and by causing CI can spread into laboratory populations^14^. As creating stable lines of *Wolbachia* in *Anopheles* is very challenging, other studies have investigated the effect of *Wolbachia* on malaria parasites by directly infecting adult *Anopheles* mosquitoes with the endosymbiont; this process differs from stable transinfections as it does not result in the transmission of *Wolbachia* to the progeny. The effects of these non-stable *Wolbachia* transinfections on *Plasmodium* are not ubiquitous and vary depending on the combination of endosymbiont strain, mosquito species, *Plasmodium* species and ambient temperature^15–18^. Therefore, the use of *Wolbachia* for malaria control may be more challenging than for arboviruses.

In natural populations, *Anopheles* malaria vectors were thought not to be infected with *Wolbachia*. However, recent studies indicate that this endosymbiont is present at low density in several African malaria vector populations^19–21^ and its presence is negatively correlated with malaria parasite^21,22^. Additionally, experimental infections with *Plasmodium falciparum* show a protective effect of the endosymbiont against the parasite, suggesting a possible role for natural *Wolbachia* strains in limiting vector competence^21^. Thus, there could be potential to use *Wolbachia* for malaria control, but given the apparent greater complexity of its effects in laboratory systems, there is a need to further elucidate its impacts on vector–parasite interactions under a range of natural, ecologically-variable conditions.

Studies of natural systems are important because a prominent determinant of *Wolbachia*-induced phenotypes is the co-evolutionary history between endosymbiont and insect host species. Indeed, natural infections might evolve towards mutualism over time^23^, showing generally lower *Wolbachia* densities and less pronounced pathogen inhibition^24^ than in artificial infections generated in the laboratory. Two studies investigated the role of natural *Wolbachia* infection by curing endosymbiont infections in *Aedes* and *Culex* species with antibiotics and observing changes in susceptibility to avian malaria parasites. In both cases (*Ae. fluviatilis* and *Cx. pipiens*), antibiotic treatment led to a reduced susceptibility to avian malaria parasites (*P. gallinaceum* and *P. relictum*, respectively)^25,26^. This finding was counter to expected protective effects of *Wolbachia* against malaria infection. However, curing *Wolbachia* in *Cx. pipiens* can lead to reduced survival once mosquitoes are infected with *P. relictum*, suggesting that natural endosymbiont infections can also indirectly play a role in the vector host transmission ability^27^. In summary, the specificities of these tripartite host–parasite and endosymbiont associations in natural systems are poorly understood.

Given the risk of effects that are opposite to those desired from interventions, it is critical to better understand the scope of evolutionary modification of these tripartite interactions. In particular, anticipation of how *Wolbachia*-infected insects released in the field may respond to natural selection requires knowledge of the factors that influence *Wolbachia*-induced phenotypes. This knowledge is needed to forecast the evolution of artificial infections and their associated impacts on host physiology and vector competence^19,22^. Investigation of these interactions in natural systems may also help resolve the otherwise difficult interpretation of conflicting results reported from artificial combinations used in the laboratory^28^.

Avian malaria is a promising natural model for the study of endosymbionts on parasite transmission^26^. Avian malaria includes apicomplexan blood parasites from the genera *Plasmodium*, *Haemoproteus* and *Leucocytozoon*^29^. The study of avian malaria is more tractable than that of human malaria, as it has a wider geographical distribution, does not pose a risk to human health and the investigation of the vertebrate–host epidemiology involves fewer ethical and practical constraints. Because avian malaria is transmitted by diverse vector taxa, this system can shed light on the contribution of vector genotype and species to *Wolbachia* phenotypes. Its vectors also include members of further groups of medical importance, for example *Culicoides* biting midges and *Simulium* black flies^30–32^. At present, natural *Wolbachia* infections have been found in some *Culicoides* populations^33^, in one African black fly population^34^ and in mosquito vectors of avian malaria^26^.

Among these groups, *Leucocytozoon*-transmitting black flies are ideally suited for studying long-term tripartite interactions for several reasons. Around 98% (2101 species) of the world’s black fly species feed on avian or mammalian hosts^35^, and many of these species are believed to transmit blood-borne pathogens^36^. Black flies are the predominant arthropod vector for almost all *Leucocytozoon* species^37^ and usually show a very high prevalence^30,32^ and diversity^32,38,39^. Black flies feed on a diverse range of avian species^37^, which vary greatly in their vulnerability to avian malaria. Indeed, *Leucocytozoon* and other apicomplexan parasites cause little pathogenicity to birds that have evolved alongside them, but are fatal to newly exposed species^40^. Black flies have been understudied vectors, in part because morphological identification cannot always resolve taxonomy. We addressed this difficulty in the *Simulium* genus^31,40^ by developing a new, practical and affordable protocol based on a restriction fragment length polymorphism (RFLP).

Here we establish a study system that exploits the diversity of black fly vector species at a single site in Scotland to investigate vector species-specific associations between *Wolbachia* and *Leucocytozoon* infection. This tripartite study of endosymbiont, vector and parasite further benefits from parallel investigation of avian malaria infection in an avian host, blue tit (*Cyanistes caeruleus*) at the same location^41^. Furthermore, we determined *Leucocytozoon* diversity and vector host specificity by sequencing and analysing the phylogeny of several identified parasite haplotypes. We found that throughout the transmission season the black fly population in this area was infected both with *Leucocytozoon* (25%) and *Wolbachia* (25%). By determining the vector species composition over the transmission season, we show that in the presence of *Wolbachia* and *Leucocytozoon* prevalence varies depending on the vector host species. This novel finding suggests that *Wolbachia* infection in black flies interacts with avian malaria transmission in a vector species-specific manner. This opens new opportunities for understanding the ecology and evolution of native endosymbiont infections and vector–pathogen interactions in a natural system.

## Results

### Scottish black fly population diversity

**Figure 1:**
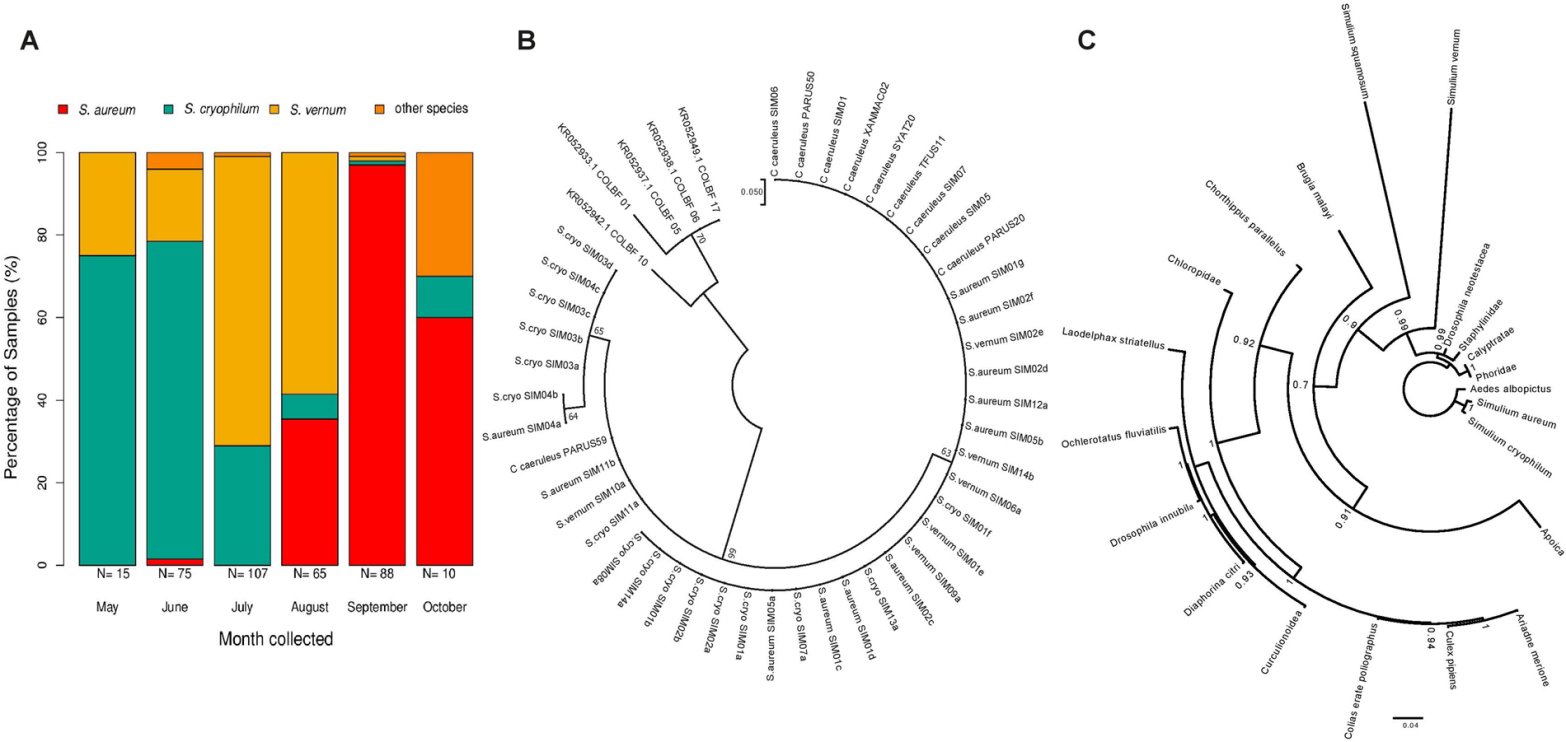
Species composition of the most abundant *Simulium* species over the season and phylogenetic analysis of the *Leucocytozoon* haplotypes detected in each species. (**A**) The percentage of each *Simulium* species collected each month, based on RFLP analysis and identification. N = X at the bottom of the x-axis indicates the total number of samples collected per month. (**B**) Phylogenetic analysis of all *Leucocytozoon* sequences from black flies and blue tits. Branches are labelled based on the infected host (*Simulium* species or *C. caeruleus*) and which parasite haplotype they are grouped in (Supplementary Table S1). (**C**) Phylogenetic analysis of *Wolbachia* MLST sequences identified in the three main black fly species in relation with other *Wolbachia* strains.

Morphological identification cannot reliably resolve taxonomy in the *Simulium* genus^32,42^. Thus we developed a new protocol based on a restriction fragment length polymorphism (RFLP) assay to determine black fly species. First, we determined the sequence of the *cytochrome C oxidase I* (*COI*) gene in a subset of samples where 4 different species were identified. Next, we selected 2 restriction enzymes to produce a unique digestion pattern for each species (Supplementary Figure S1, see Methods for details). The validity of the technique was confirmed by sequencing 3 samples per species after the RFLP assay identification. This relatively quick and efficient assay allowed us to determine the species of 370 sampled black flies. Three species dominated the black fly community at Scottish forest sites from May to October 2015 (Table 1): *Simulium aureum*, *S. vernum* and *S. cryophilum*, with 4 additional species making up the remainder (Table 1). There was pronounced seasonal variation in black fly species composition (Fig. 1A). *Simulium cryophilum* was predominant at the start of the season (May–June), then replaced by *S. vernum* in mid-season (July–August), and by *S. aureum* in the late season (August–October). We conducted a phylogenetic analysis using the *COI* gene to determine the genetic relationship between these black fly species. Including species from Africa (*S. squamosum*) and North America (*S. vittatum*) as outgroups, the species we collected were most closely related to the North American black fly species (Supplementary Figure S2).

**Table 1:** Total number of each species identified using the RFLP method from 370 successful extractions. Percentage of each species is indicated in brackets.

Species	Total observed
*S. aureum*	115 (31%)
*S. vernum*	137 (37%)
*S. cryophilum/S. urbanum*	110 (29.6%)
*S. verecundum*	3 (0.9%)
*S. variegatum*	1 (0.3%)
*S. argyreatum/S. variegatum*	1 (0.3%)
*S*.* intermedium*	3 (0.9%)

### Avian malaria and Wolbachia prevalence in black flies

We found that 25% (93 out of the 370) black flies collected were infected by *Leucocytozoon*. Sequencing of the *cytochrome B* (*cytB) Leucocytozoon* gene in 33 positive samples confirmed the presence of the parasite (accession numbers MG649331-MG649363). We separated the head/thorax section from the abdomen of 21 specimens and tested these tissues separately, with the aim of distinguishing between transmittable parasites in the mouthparts and those still developing in the midgut. We detected infections in both tissues in all samples, suggesting that the black flies harboured transmission-stage parasites. Sequence alignments identified 14 haplotypes with specific matches to a public database of avian malaria parasites, MalAvi^43^ (Supplementary Table S1). Four of these haplotypes were also identified in the blue tits that were sampled in the same season and locality^41^ (Supplementary Table 1), suggesting that black flies are responsible for local transmission to this bird species. To confirm this hypothesis, we conducted phylogenetic analysis comparing the black flies’ and blue tits’ *Leucocytozoon* haplotypes, using *Leucocytozoon* sequences from black flies collected in Colorado as outgroups^32^. The analysis indicated that *Leucocytozoon* haplotypes in Scottish black flies cluster with those in the blue tit, but are different from those derived from Colorado black flies (Fig. 1B).

We then tested black flies for the presence of *Wolbachia* using *Wolbachia* surface protein (*wsp*) gene-specific primers. Ninety-two *Wolbachia*-infected samples (24.8%) were detected by PCR. Sequencing *wsp* in 20 positive samples further confirmed the presence of *Wolbachia* (accession numbers MG493280-MG493291). To further characterize the identified strains, we performed multilocus sequence typing (MLST) for *Wolbachia*. We sequenced the standard five ubiquitous genes (*gatB*, *coxA*, *hcpA*, *fbpA*, and *ftsZ*) from a single individual for each of the 3 vector species and identified new *Wolbachia* alleles (accession numbers MG649364-MG649378). Amongst these, we found a small number of nucleotide sites that suggested polymorphism (1 in *S. aureum-hcpA*, 3 in *S. cryophilum-ftsZ*, 4 in *S. vernum-ftsZ* and 10 in *S. vernum-coxA*) (Supplementary Table S3) and potentially multiple infections. To exclude the influence of the polymorphic sites on the phylogenetic reconstruction, we replaced polymorphic sites with Ns. Phylogenetic analysis infers the *Wolbachia* strain in *Simulium vernum* to be a sister lineage to the strains from a great range of different host species including from *Simulium aureum* and *Simulium cryophilum*, which in turn cluster together (Fig. 1C). Phylogenetic analysis including single nucleotides in polymorphic sites obtained from cloned sequences gave a very similar phylogeny (Supplementary Figure S3).

### Leucocytozoon and Wolbachia prevalence variation during the season

**Figure 2:**
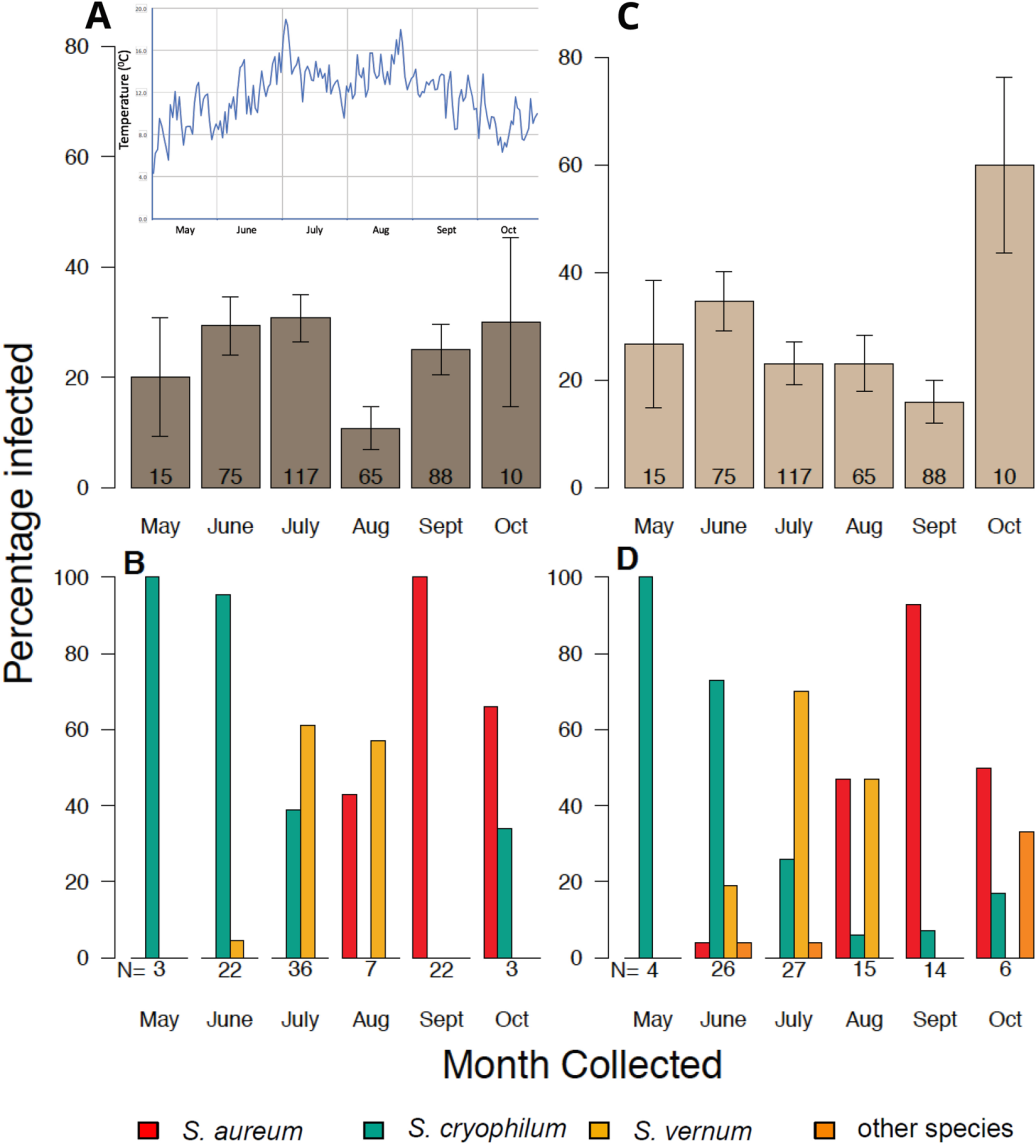
Variation in *Leucocytozoon* and *Wolbachia* infectivity of black flies based on month collected and black fly species identified. (**A**) shows the percentage of black fly individuals which tested positive for *Leucocytozoon* per month. On top, the average daily temperature between May and October from the nearest Met Office weather station (Ardtalnaig) taken between 9.00 am-9.00 pm. (**B**) shows the proportion of black fly species infected with *Leucocytozoon* among the overall infected samples per month. (**C**) shows the percentage of individuals which tested positive for *Wolbachia* per month and (**D**) shows the proportion of black fly species infected with *Wolbachia* among the overall infected samples per month. Figures in bars of A and C indicate the total number of samples collected and tested per month. N = X shown below B and D indicate the number of samples positive for either *Leucocytozoon* or *Wolbachia*, respectively. Error bars in A and C represent the standard errors of the mean.

A general linear mixed effects model was used to test for associations between avian malaria prevalence within vectors, black fly species and sampling month. Parasite prevalence changed significantly over the summer months (LRT: X^2^ (5) = 12.06, *p* = 0.03), with infection rates in August being lower than in any other trapping month (Fig. 2A and Supplementary Table S4). August was the month with the highest daily mean temperatures in the study area (Fig. 2A), thus further studies are needed to understand if ambient temperature is associated with parasite prevalence in the vector hosts. As malaria infection prevalence was not significantly affected by the interaction between month and black fly species (LRT: X^2^ (10) = 7.2, *p* = 0.67), the infection prevalence variation over the season does not seem to be black fly species-specific, although larger sample sizes would be needed to confirm this observation (Fig. 2B and Supplementary Table S4).

A general linear mixed effects model was used to test whether endosymbiont prevalence varied between black fly species and the month they were collected. *Wolbachia* prevalence did not vary with sampling month (LRT: X^2^ (5) = 8.1, *p* = 0.15), or between black fly species (LRT: X^2^ (3) = 3.7, *p* = 0.29) (Fig. 2C,D and Supplementary Table S4).

### Wolbachia presence correlates with Leucocytozoon prevalence in a vector species-specific manner

**Figure 3:**
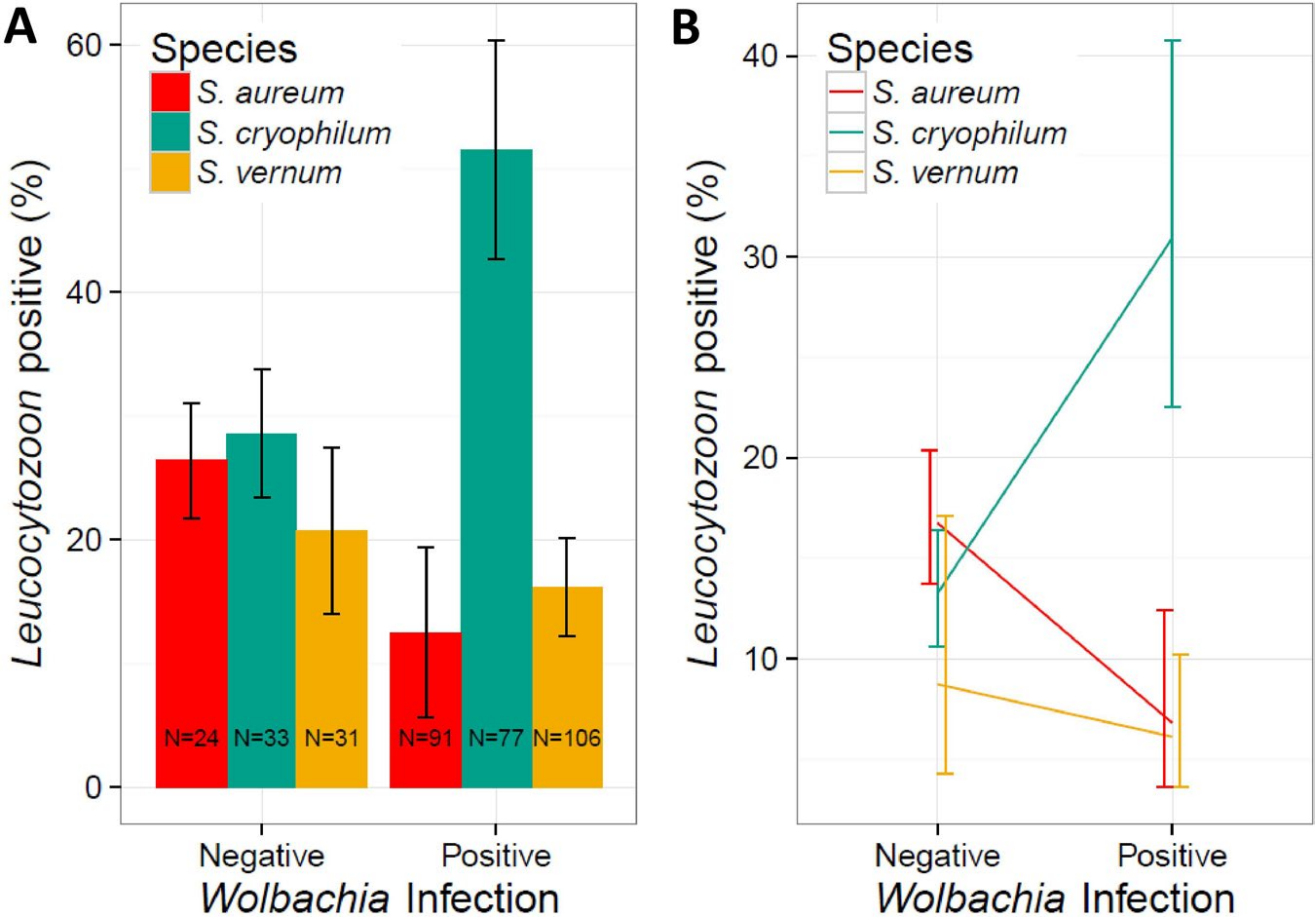
Variation of *Leucocytozoon* prevalence in black flies in the presence of *Wolbachia*. (**A**) The proportion of *Leucocytozoon*-infected black flies in the presence and absence of *Wolbachia* based on PCR results for each of the three major black fly species identified, *S. aureum*, *S. vernum* and *S. cryophilum*. Error bars represent the standard error calculated from the mean value of each data point and N = X represents the number of samples in each set. (**B**) *Leucocytozoon* prevalence in the presence and absence of *Wolbachia* for each black fly species based on the coefficients of the significant interaction between *Wolbachia* and black fly species from the best fitting model.

A generalised linear mixed model was used to test for associations between *Wolbachia* and malaria infection in individual black flies (Fig. 3A), while controlling for variation due to sampling month and black fly species. This indicated that malaria parasite presence was significantly influenced by the interaction between black fly species and *Wolbachia* infection status (LRT: X^2^ = 8.71 (2), *p* = 0.013). Specifically, the presence of *Wolbachia* in *S. cryophilum* was associated with an almost 3-fold increase in *Leucocytozoon* infection probability. In contrast, in *S. aureum* parasite prevalence was ~50% lower in flies infected with *Wolbachia*, while in *S. vernum*, *Leucocytozoon* prevalence was approximately similar in black flies with and without *Wolbachia* (Fig. 3B).

## Discussion

Our analysis of black fly populations reveals for the first time the presence of seasonally stable *Wolbachia* and avian malaria infection in 3 *Simulium* species. The presence of *Wolbachia* correlated with the probability that vectors were infected with the malaria parasite *Leucocytozoon*, but the association between *Wolbachia* and *Leucocytozoon* varied between black fly species. Whilst *Wolbachia* was negatively associated with reduced malaria prevalence in *S. aureum*, it was positively associated with malaria prevalence in *S. cryophilum*, and uncorrelated in *S. vernum*. MLST and phylogenetic analysis suggests that each black fly species harbours a different *Wolbachia* strain/s, which could potentially explain the diversity of *Plasmodium* associations observed. These results unveil the complexity of the tripartite interactions between the invertebrate vector, the parasite and the endosymbiont, and highlight the potential pitfalls of extrapolating general effects from laboratory experiments.

The observed mean *Wolbachia* prevalence of 25% is difficult to reconcile with *Wolbachia*-induced reproductive manipulation such as CI, as this phenomenon would be expected to lead to endosymbiont fixation^4^. However, reproductive manipulation is not always associated with *Wolbachia*, as CI can be complete (e.g. in mosquitoes)^44,45^, weak (e.g. in some *D. melanogaster* strains)^46,47^ or even absent (e.g. in *D. simulans*)^48^. Indeed, *Wolbachia* can persist even in the absence of reproductive parasitism by providing a fitness advantage to its insect host; this is the case in *Drosophila simulans*, where *Wolbachia* protects the host from pathogenic viruses^49^. Alternatively, persistence of *Wolbachia* in these black fly populations could occur through continuous loss and gain over generations. For example, it has recently been shown that *Wolbachia* can be horizontally transmitted through plants^50^.

The black fly species-specific associations between *Wolbachia* and avian malaria infection might depend on *Leucocytozoon*, *Wolbachia* and the vector genotypes. As *Leucocytozoon* haplotypes were shared between different black fly species, we might exclude that parasite genetics influences these interactions. In contrast, MLST and phylogenetic analysis identified specific *Wolbachia* strains in each black fly species. Therefore, the vector and endosymbiont genotype could play a role in the observed associations. Nevertheless, the outcome of this tripartite interaction is probably shaped by a combination of *Wolbachia* genotype, vector genotype and behavior, and environmental conditions. For example, although *S. cryophilum* and *S. aureum* strains appear to be closely related, they showed opposite associations with the parasite indicating that factors other than *Wolbachia* genotype might be responsible. The immunity and physiology of the three black fly species in the presence of *Wolbachia* could vary, leading to different phenotypes. Alternatively, environmental factors could have shaped the nature and magnitude of *Wolbachia* impacts on malaria infections in black flies. For example, the black fly species sampled here show a distinct seasonal phenology, and are thus exposed to somewhat differing environmental conditions. Diet and temperature are known to alter *Wolbachia* density in the host^51,52^, which can ultimately affect its phenotype^53^; although exceptions where *Wolbachia* density is not associated with CI - e.g. in *Drosophila*^48,54^, nor with malaria parasite interference^15,16^ exist. Notably, changes in temperature alter the pathogen interference phenotype of *Wolbachia* against the rodent malaria parasite *Plasmodium yoelii* in the mosquito *An. stephensi*^17^. Therefore, it is possible that seasonal temperature variations are responsible for the differential associations between *Wolbachia* and avian malaria observed in black flies that emerge during different periods between May and October. Indeed, during the study period the daily mean temperature ranged from 4.3 °C to 19 °C, which could have influenced *Wolbachia*–parasite interactions. While previous studies have investigated the effect of temperature under controlled conditions in the laboratory, the novel system described here could allow these effects to be investigated in a natural, seasonally variable environment.

Previous reports show that an avian malaria system consisting of *Cx. pipiens* infected with *P. relictum* is well suited for the study of *Wolbachia* phenotypes^26,55^. Nevertheless, as *Cx. pipiens* mosquitoes are all infected with *Wolbachia* in nature, there is little opportunity to investigate naturally occurring variation and how it relates to parasite infection. In contrast, this *Leucocytozoon* system permits the study of multiple vector host species in the presence and absence of *Wolbachia*, like the situation observed in different populations of *Anopheles* malaria vector species in Burkina Faso and Mali, which vary in their *Wolbachia* infection rate^19,21,22^. Indeed, an important asset of this avian malaria model is the high likelihood of sampling insects with co-infections of *Wolbachia* and transmission-stage malaria parasites. In our study, the identification of malaria infections in black flies in both the abdomen and head might suggest that these vectors harboured infective sporozoite stages in their salivary glands. The *Leucocytozoon* extrinsic incubation period (i.e. the cycle in the invertebrate host) has approximately the same duration as the black fly gonotrophic cycle (4 to 5 days)^56,57^; therefore, females that were infected in their previous blood meal would most likely have developed sporozoites by the time they seek a second host. Detection of the parasites in the saliva or experimental infections could confirm that the collected black flies can transmit *Leucocytozoon*. The considerably shorter extrinsic incubation period of *Leucocytozoon* compared to *Plasmodium*^56^ or *Haemoproteus*^58^ could be another advantage of this model system.

At our study site in Scotland, *Leucocytozoon* prevalence in black flies was lower than previously reported in other populations in Sweden and Colorado^30,32^. However, prevalence may have been overestimated in the Swedish population as this was based on sampling black flies that still had blood in their guts. Therefore, black flies in the Swedish study could have merely fed on infected host blood, but were not actually infected themselves^30^. In Colorado, host-seeking black flies were analysed as in this study, but in pools of 5 individuals rather on an individual basis as done here. Thus the estimate of 46% prevalence could be an overestimate^32^. Furthermore, *Leucocytozoon* prevalence might vary between different areas. Although parasite prevalence did not vary across the three main black fly species collected, there was a non-significant trend of somewhat higher prevalence in *S. cryophilum* (35%) compared to the other two main species (*S. vernum*: 19%, *S. aureum*: 23%). Larger sample sizes are required to conclusively test for small, vector-species-specific differences in malaria infection rate. Notably, as we did not find clear associations between *Leucocytozoon* haplotypes and black fly species, the three main vector species might share the same avian hosts, with no distinct host species preferences. This hypothesis is in accordance with previous work, where no clear patterns of avian host species and *Leucocytozoon* haplotypes were identified in black flies^32^, but in contrast with a study where such associations were observed^30^. Characterisation of the specific avian hosts of these black flies by blood meal identification would be needed to better understand the degree of vector–host preference in this system and clarify whether the black fly species have a similar vectorial capacity.

While *S. aureum* has been well characterised in the UK by morphological identification^59^, there are limited reports of species determination of *S. vernum* and *S. cryophilum* by this method. Here we developed a RFLP assay to quickly determine the species of morphologically indistinguishable black flies without sequencing them. This method appears to be robust enough to characterise the populations in the studied area, although the application to other black fly populations needs to be investigated. For example, it could be possible to use this RFLP species identification assay on other black flies upon: i) sequencing the *COI* gene of local species ii) the determination of the expected digestion profile, and iii) incorporation/change of another restriction enzyme if required.

In conclusion, our identification of natural *Wolbachia* infections in vectors of avian malaria, together with the development of a new molecular assay that rapidly identifies black fly species, will allow more in-depth studies of these tripartite interactions in nature. The high prevalence and diversity of *Leucocytozoon* in different black fly species, the possibility of obtaining infected insects, and the reduced ethical issues compared with the study of human malaria make this system a useful, cost-effective and practical model for the study of pathogen–host interactions and disease transmission. At a time when *Wolbachia* is already undergoing field trials for use as a biocontrol against dengue^11^ and has been proposed for use in malaria control^14,22^, understanding the longer-term evolution and ecological dynamics of *Wolbachia* phenotypes in insect vectors is timely.

## Materials and Methods

### Sampling time and location

Black fly collection was conducted at the Scottish Centre for Ecology and Natural Environment (SCENE) from the 16th of May to 15th of October 2015. Two main areas were selected for sample collection: the first is a woodland area situated approximately 750 meters west of the SCENE which was named “Hole” (GPS coordinates: 56°7′36. 3′′N; 4°37′24. 7′′W). Hole area extends for about 0.1 square kilometres, at a maximum altitude of 75 meters on the sea level, and is dominated by oak. The second area was part of Cashel forest (GPS coordinates: 56°6′38.3′′N; 4°34′37.2′′W), located 3.7 kilometres southeast of SCENE. Cashel Forest is an area managed by the Royal Scottish Forestry Society; it extends over 12.14 square kilometres, reaches a maximum altitude of 80 meters on the sea level and its vegetation is mainly constituted by oak and birch forest. On its west side, it borders farmlands that are mainly used as sheep pastures.

Hole-nesting songbirds at both sites have high prevalence of avian malaria^41^. Specifically, blue tits are typically infected with avian malaria during early nestling stages, with *Leucocytozoon* prevalence detectable in blood in most two-week old nestlings^41^. For our study, avian *Leucocytozoon* sequences originated from blue tits sampled at the same two sites in the same study period. We used samples from 10 blue tits, collected in June 2015, and spread evenly across the sites (Hole: n = 5; Cashel: n = 5) and age groups (nestlings: n = 5; adults: n = 5). The study involving blue tits was carried out in accordance with the recommendations of UK Home Office (project licence: 70/7899 to BH), Scottish Natural Heritage (52463 to BH) and British Trust for Ornithology (Scientific C licence to BH). The protocol was approved by the UK Home Office.

### Sampling methods and sampling effort

Eight Centers for Disease Control and Prevention miniature light traps (CDC-LC, mod. 512, John W. Hock Company) were used to sample black flies across the 2 study sites: 4 traps were placed in Hole and 4 in Cashel Forest. Traps were positioned 1.5 meters from ground level hanging from a tree branch; their location remained unvaried throughout the study. Light traps were active overnight from 18:00 to 9:00. CO_2_ is commonly used as bait for hematophagous insects, as it constitutes an important host-localization cue for a broad range of species^60^. Light traps were baited with insulated dry-ice containers (John W. Hock Company). The containers were filled for half of their volume with dry ice pellets and this ensured constant release of CO_2_ for a period of 12 hours. The baiting could not be performed on every collection night due to difficulties in transport and storing of the dry ice. Over the 5 months period a total of 159 light trap collections, 36 of which baited with CO_2_, were performed. Four light traps collections per site were carried out every 48 hours. When dry ice was available, 2 of the 4 traps per site were baited with CO_2_. Rotation of the baiting was conducted each night to prevent trap location bias. Once retrieved, the collection tubs of the light traps and baited light traps were stored in a freezer for a period of ~30 minutes to kill the specimens. Black flies were then separated from other insects that were not of interest using a stereo dissection microscope (Nikon SMZ-745T) and preserved in 95% ethanol at −20 °C.

### DNA extraction, *Leucocytozoon* and *Wolbachia* detection

Black fly DNA was extracted using a kit (DNeasy whole-blood extraction kit, Qiagen) as per manufacturer’s instructions and eluted in 50 µl of nuclease-free water. To test for *Leucocytozoon* parasites infections, we examined the black fly DNA using a nested polymerase chain reaction (PCR) following an established method^42^, that amplified a 479 bp fragment of the *Leucocytozoon* mitochondrial cytochrome B (*cytB*) gene. PCRs were performed in a 20 µl reaction mixture using 1× GoTaq universal PCR master mix (Promega), ~100ng of black fly DNA and 0.6 µM of each primer. Out of 93 positive samples, 33 were purified by kit (Wizard SV Gel and PCR Clean-Up System, Promega) and sequenced using either the forward or reverse PCR primer (Eurofins Genomics). Initial testing of samples involved dissecting the flies by their head and thorax or their abdomen to examine whether the parasite was in an infectious stage (sporozoites are present in the salivary glands in the thorax and oocysts are present in the midgut). We amplified DNA from the separately extracted parts to determine where *Leucocytozoon* was present. Only 2 of the 372 black flies were found to contain a blood meal when examined under the microscope.

To test for *Wolbachia* presence, PCRs were set up to amplify a 546 bp fragment of the *Wolbachia* surface protein (*wsp*) gene following an established method^61^. The PCR was performed in a 20 µl reaction mixture using 1× GoTaq universal PCR master mix (Promega), 1 µM of each primer and ~100ng of template DNA. Amplified DNA from the PCRs was visualized on a 1.5% agarose gel using SyberSafe (10%; Invitrogen; 10 μl per 100 μl of gel). Twenty positive samples were purified by kit (Wizard SV Gel and PCR Clean-Up System, Promega) and sequenced using either the forward or reverse PCR primer (Eurofins Genomics).

### *Wolbachia* multilocus sequence typing (MLST)

To amplify MLST genes (*gatB, coxA, hcpA, fbpA*, and *ftsZ*) we used a nested PCR approach. Initial PCR was conducted using external primers with M13 adaptors (M13 forward: TGTAAAACGACGGCCAGT M13 reverse: CAGGAAACAGCTATGACC) added to each primer: gatB_F2: CAGATAACNCARTTYTTYGARCC, gatB_R2: ATTGTTCCATCNACDATRAARTC; coxA_F2: GGAGGATTYGGNAAYTGGTTYGT, coxA_R2: CCACCCCACATNGTNGCDATCCA; fbpA_R2: TTACCGCCACCYTGYTTDATYTC, fbpA_R2: TTACCGCCACCYTGYTTDATYTC; hcpA_R2: ACATACTGNACRTCRTCRTTRTC, hcpA_R2: ACATACTGNACRTCRTCRTTRTC, ftsZuniF: GG(CT)AA(AG)GGTGC(AG)GCAGAAGA ftsZuniR: ATC(AG)AT(AG)CCAGTTGCAAG^62,63^. PCRs were performed in a 20 µl reaction mixture using 1 × GoTaq universal PCR master mix (Promega), 0.5 µM of each primer and ~100 ng of template DNA; after initial denaturation at 95 °C 15 mins, 50 cycles at 95 °C 15 secs, 55 °C 30 secs and 72 °C 1 min were performed followed by 10 mins 72 °C final extension. Nested PCR was conducted using standard *Wolbachia* MLST primers following establish protocol^61^. PCRs were performed in a 20 µl reaction mixture using 1× GoTaq universal PCR master mix (Promega), 1 µM of each primer and 2 µl of initial PCR (1/10). Five genes for individual black fly for each of the three species were purified (Wizard SV Gel and PCR Clean-Up System, Promega) and sequenced using both the forward or reverse primers (Eurofins Genomics). In 4 out of 15 sequenced genes (1 in *S. aureum*, 1 in *S. cryophilum*, 2 in *S. vernum*) (Supplementary Table S3) some polymorphic nucleotides were identified. In these cases, we cloned the amplified fragments (TA cloning kit, Invitrogen). In two cases (*S. aureum-hcpA, S. vernum-ftsZ)* polymorphism could not be called reliably even after cloning. Sequences were deposited both in Genbank (accession numbers MG649364-MG649378) and *Wolbachia* MLST database (https://pubmlst.org/wolbachia/).

### *Simulium* species identification by restriction fragment length polymorphisms (RFLP)

PCR was set up to amplify a 709 bp fragment of *Simulium* DNA from the *cytochrome C oxidase I* (*COI*) gene as previously described^64^. The PCR was performed in a 20 µl reaction mixture using 2× GoTaq universal PCR master mix (Promega), 0.3 µM of each primer and ~100 ng of template DNA. First, species were assigned in 15 samples where a full read of the *COI* gene fragment was obtained and aligned using BLAST (accession numbers MG599009-MG599023). Four species were identified by initial sequence analysis (*S. vernum*, *S. aureum*, *S. intermedium* and *S. cryophilum*). A few samples were classified as *S. urbanum* (GenBank: KP861204.1) by BLAST analysis, but as these sequences were 100% identical to *S. cryophilum*, for simplicity these samples are referred to as *S. cryophilum* throughout this report. Then, a protocol was designed using restriction fragment length polymorphisms (RFLP) to identify the species of all the black fly samples tested. Sequences were aligned using Clustal Omega and restriction enzymes were selected using the NEBcutter V2.0 (http://nc2.neb.com/NEBcutter2/) based on an appropriate number of cut sites which resulted in unique sizes of fragments produced per species (as shown in Supplementary Table S2). All samples (8 out of 370) which were not digested by restriction enzymes (potentially being *S. intermedium*, Supplementary Figure S1) were sequenced and additional species were found. Three of each identified species by RFLP were also sequenced to confirm the protocol was accurate. Each reaction was set up under the following conditions: 15 minutes at 37 °C, 20 minutes at 65 °C for enzyme deactivation, and held at 4 °C. Each 20 µl reaction contained 2.0 µl of PCR product (1/10 of *COI* gene PCR), 4 units of NheI, 4 units of NsiI (both enzymes supplied by NEB), and 1× NEB buffer 2.1 (50 mM NaCl, 10 mM Tris-HCl, 10 mM MgCl2, 100 μg/ml BSA, pH 7.9 at 25 °C). Samples were analysed on a 2% agarose gel (2 g agarose powder in 100 ml 0.5% Tris-borate EDTA buffer). Species were determined based on the expected fragment sizes shown in Supplementary Figure S1.

### Phylogenetic analysis

Based on the acquired sequences, phylogenetic analysis was performed to determine sequence similarities between the black species and *Leucocytozoon* species. *Simulium* sequence IDs were confirmed using NCBI BLAST and were aligned using Clustal Omega. Sequence divergences were calculated using the General Time Reversible (GTR + G) model^65^. A maximum likelihood tree using 500 bootstrap replicates of GTR + G distances was created to provide a graphic representation of the patterning of divergences among the sequences obtained from the samples. This was then refined to show a single representation for each identified species based on the top BLAST matches. To determine the relationships of the different species of black fly found around Loch Lomond, sequences for the COI gene of three species from other studies were included. Two species, *S. venustum* and *S. vittatum* are commonly found in the Nearctic^66^ and one species, *S. squamosum*, is the species that was reported to carry *Wolbachia* in Ghana^34,67^. *Leucocytozoon* sequence IDs were confirmed using the MalAVI database (Supplementary Table S1). Sequence divergences were calculated using the Tamura 3-Parameter model^65^. A maximum likelihood tree using 500 bootstrap replicates of Tamura 3-Parameter distances was created to provide a graphic representation of the patterning of divergences among the sequences obtained from the samples. Sequences from Colorado, USA^32^, were used to indicate phylogenetic divergence.

For MLST phylogenetic analysis the five genes were aligned separately in CLC Genomics Workbench (‘very accurate alignment’, gap open cost 10, gap extension cost 1, end gap cost cheap). Ends were trimmed to reduce missing data, end gaps and missing data coded as N, and genes were concatenated for a total of 2082 bp. The final matrix consisted of 2082 bp (402 bp of *coxA*, 429 bp of *fbpA*, 435 bp of *ftsZ*, 369 bp of *gatB*, 447 bp of *hcpA*). The best-fit model of evolution, allowing up to one partition per gene, was inferred using Partitionfinder v2.1.1^68^ and the best scheme selected by AICc. A Bayesian phylogenetic tree was inferred in MrBayes v3.2.6^69^, with gaps as informative, no outgroup, variable rate prior, and unlinked characters sets (unlink state freq, revmat, shape, pinvar, tratio); all other parameters at default. The four chain mcmc was run for 25 million generations sampled every 500 generations and 25% of generations were eliminated as burn-in before summarising the consensus tree. A three partition scheme was inferred to be the best fit to the data with TVM + I + G for *coxA*, TVM + G for *fbpA*, and GTR + G for a partition including *ftsZ*, *gatB*, and *hcpA*. A three partition scheme was inferred to be the best fit to the data with TVM + I + G for *coxA*, TVM + G for *fbpA*, and GTR + G for a partition including *ftsZ*, *gatB*, and *hcpA*.

### Statistical analysis

Statistical analysis was performed using three general linearised mixed-effects model (GLMM) using R statistical software^70^ and the lme4 package^71^. One investigated the significance of several explanatory variables on the response variable *Wolbachia* prevalence in the black fly population. The other two analysed *Leucocytozoon* prevalence in the vectors as the response variable, including or excluding *Wolbachia* as a fixed effect. Each model used binomial distribution and any significant explanatory variables and significant interactions were determined using log likelihood ratio tests (LRT). In each model, ‘Site’ (the two trapping locations ‘Hole’ and ‘Upper Cashel’) and ‘DNA Yield’ (the concentration of DNA in ng/µl from each extraction, based on Nanodrop analysis) were fitted as random effects, while the categorical variables of ‘month collected’ (May, June, July, August, September and October), ‘black fly species’ (3 main species – *S*. *vernum, S. aureum* and *S. cryophilum*), and ‘trap type’ (light trap or baited CO_2_ light trap) were all fixed effects. Additionally, in one model where *Leucocytozoon* prevalence was the response variable, *Wolbachia* was also included as a fixed effect. This latter model used a binomial distribution and incorporated fixed and random effects, and an interaction between black fly species and *Wolbachia*. This was determined to be the best fitting model by examining the log-likelihood ratio and the residual deviance of the model.

## Electronic supplementary material

Supplementary material
