# Supplementary material for: Vector species-specific association between natural Wolbachia infections and avian malaria in black fly populations
Source: Sci Rep. 2018 Mar 8;8:4188. doi: 10.1038/s41598-018-22550-z (PMC5843599; doi:10.1038/s41598-018-22550-z)
Supplement: Supplementary file 1 — Supplementary material [file 41598_2018_22550_MOESM1_ESM.pdf]

Supplementary Material

Vector species-specific association between natural *Wolbachia* infections and avian malaria in black fly populations

Luke Woodford[1,2], Giovanni Bianco[1], Yoana Ivanova[1], Maeve Dale[1], Kathryn Elmer[1], Fiona Rae[1], Stephen D. Larcombe[1], Barbara Helm[1], Heather M. Ferguson[1] and Francesco Baldini[1*]

[1]Institute of Biodiversity, Animal Health and Comparative Medicine, University of Glasgow, United Kingdom

[2]Department of Biomolecular Sciences, School of Biology, University of St Andrews, United Kingdom

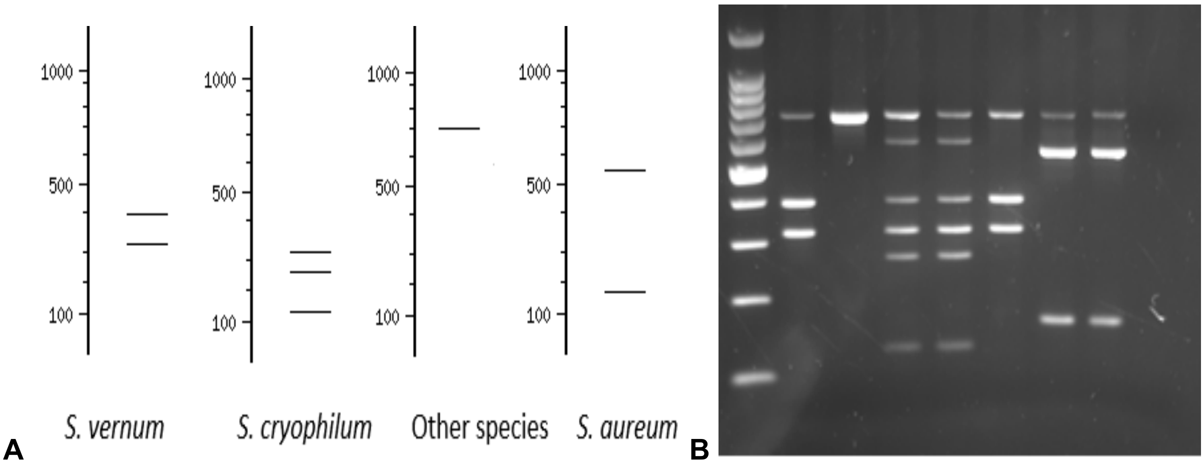

Supplementary Figure S1: A) Expected RFLP fragment sizes for each characterized *Simulium* species. Each band represents a fragment produced from the digestion. The marker column to the left of each set of fragments indicates the approximate base pair sizes. **B)** RFLP fragments shown by gel electrophoresis analysis (sample order - lane 1 = 50bp ladder, lane 2 and 6 = *S. vernum*, lane 3 = *S. intermedium* (or other), lane 4 and 5 = *S. cryophilum*, lane 7 and 8 = *S. aureum*). Some non-digested amplicon is visible in all samples, although a clear specific pattern can be determined for all species tested.

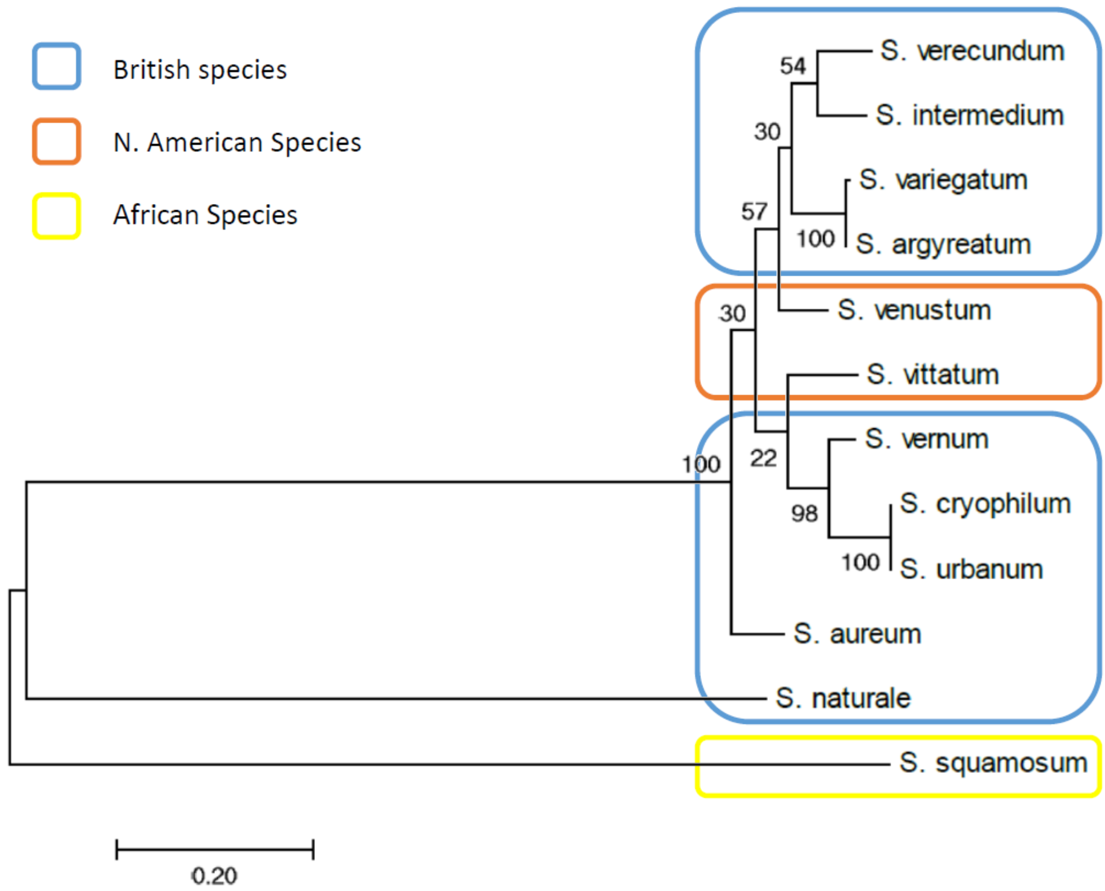

Supplementary Figure S2 - Molecular phylogenetic analysis of all Simulium species identified using Maximum Likelihood method. Phylogenetic analysis based on the Tamura 3-parameter model [1]. The tree with the highest log likelihood (-2916.5242) is shown. The percentage of trees in which the associated taxa clustered together is shown next to the branches. Initial tree(s) for the heuristic search were obtained automatically by applying Neighbor-Join and BioNJ algorithms to a matrix of pairwise distances estimated using the Maximum Composite Likelihood (MCL) approach, and then selecting the topology with superior log likelihood value. The tree is drawn to scale, with branch lengths measured in the number of substitutions per site. The analysis involved 12 nucleotide sequences. There were a total of 449 positions in the final dataset. Evolutionary analyses were conducted in MEGA7 [2].

Supplement Table S1

Best MalAvi database BLAST hits for *Leucocytozoon* haplotypes found in black flies and blue tits (*Cyanistes caeruleus*)[3] in the same season and location in Scotland.

Haplotype	Detected in	Length bp	Top MALAVI hit	E-value	% identity
SIM01	*S.cryophylum*	372	PERATE04	0	100
SIM01	*S.cryophylum*	96	PERATE04	2e-46	100
SIM01	*S.aureum*	123	PERATE04	2e-62	100
SIM01	*S.aureum*	174	PERATE0	2e-89	100
SIM01	*S.vernum*	174	PERATE04	2e-89	100
SIM01	*S.cryophylum*	162	PERATE04	7e-83	100
SIM01	*S.aureum*	96	PERATE04	2e-46	100
SIM02	*S.aureum*	111	WW6	1e-54	100
SIM02	*S.cryophylum*	82	WW6	1e-38	100
SIM02	*S.cryophylum*	297	WW6	8e-85	100
SIM02	*S.aureum*	132	WW6	4e-60	100
SIM02	*S.aureum*	120	WW6	1e-59	100
SIM02	*S.vernum*	126	WW6	6e-63	100
SIM03	*S.cryophylum*	135	NEVE01	6e-68	100
SIM03	*S.cryophylum*	102	NEVE01	1e-49	100
SIM03	*S.cryophylum*	123	NEVE01	3e-61	100
SIM03	*S.cryophylum*	123	NEVE01	3e-61	100
SIM03	*S.cryophylum*	432	NEVE01	0	100
SIM04	*S.aureum*	123	MTUR2	3e-61	100
SIM04	*S.cryophylum*	102	MTUR2	1e-49	100
SIM05	*S.aureum*	471	PARUS4	0	100
SIM05	*S.aureum*	438	PARUS4	0	100
SIM06	*S.vernum*	471	PARUS14	0	99
SIM07	*S.cryophylum*	471	PARUS21	0	100
SIM08	*S.cryophylum*	99	SERSER04	4e-48	100
SIM09	*S.vernum*	98	ZOCAP05	3e-49	100
SIM10	*S.vernum*	81	TUPHI06	7e-35	100
SIM11	*S.aureum*	141	TRPIP2	3e-75	100
SIM11	*S.cryophylum*	75	TRPIP2	7e-35	100
SIM12	*S.aureum*	267	AEMO02	5e-136	100
SIM13	*S.cryophilum*	454	COLIV04	0	100
SIM14	*S.cryophylum*	180	GAGLA06	8e-93	100
SIM14	*S.vernum*	189	GAGLA06	4e-96	100
	Cyanistes caeruleus	296	PARUS20	4e-157	100
SIM05	*Cyanistes caeruleus*	451	PARUS4	0	100
SIM07	*Cyanistes caeruleus*	178	PARUS21	9e-92	100

TFUS11	*Cyanistes caeruleus*	123	TFUS11	2e-61	100
SYAT20	*Cyanistes caeruleus*	265	SYAT20	6e-140	100
XANMAC02	*Cyanistes caeruleus*	98	XANMAC02	1e-47	100
SIM01	*Cyanistes caeruleus*	84	PERATE04	7e-40	100
PARUS59	*Cyanistes caeruleus*	221	PARUS59	1e-115	100
SIM06	*Cyanistes caeruleus*	218	PARUS14	3e-112	99
PARUS50	*Cyanistes caeruleus*	395	PARUS50	0	100

Supplement Table S2: Total cut sites and the fragment sizes produced for each Simulium species. Buffer 2.1 was considered optimal for both enzymes and the same incubation temperature was required for enzyme activity.

**Enzyme**	**Number of fragments per species**				**Buffer 2.1 activity**	**Incubation temp (°C)**
	S. vernum	*S. cryophilum*	*S. intermedium*	*S. aureum*		
NheI	I	II	-	-	100%	37
NsiI	-	-	-	I	75%	37
Cut sizes (bp)	391/318	318/263/128	709	545/164	N/A	N/A

Supplement Table S3: List of Single nucleotide polymorphisms (SNPs) identified in the *Wolbachia* MLST genes from three main black fly (*Simulium*) species.

	S. cryophillum	*S. aureum*	*S. vernum*
Gene	Single nucleotide polymorphisms		
coxA	none	none	C56T, G134A, T190C, A193G, T277C, C280T, A283G, C298T, T333C, A397G
fbpA	none	none	T88C, G129A, G340A, T350C
ftsZ	T341C, C383T, T394C	none	none
gatB	none	none	none
hcpA	none	A314C	none

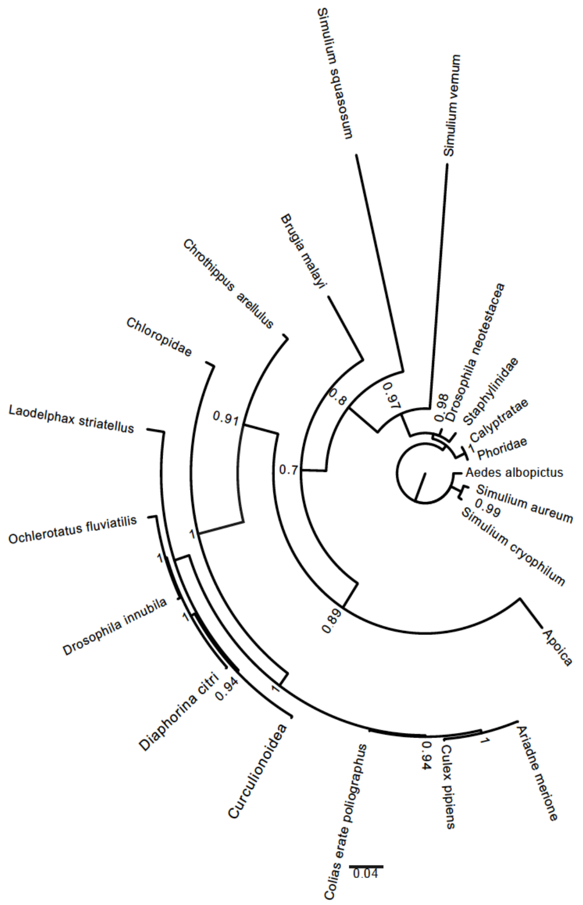

Supplementary Figure S3: Phylogenetic analysis of *Wolbachia* MLST sequences representative of individual alleles identified in the three main black fly species in relation with other *Wolbachia* strains.

Supplementary Table S4 - number of samples which tested positive for *Leucocytozoon* (L) and *Wolbachia* (W) per species (percentage of infected per species shown in brackets).

**Month**	***S. aureum***			***S. vernum***			***S. cryophilum***			**Other species**		
	Total	L	W	Total	L	W	Total	L	W	Total	L	W
May	0	0	0	3	0	0	12	3 (25)	4 (33)	0	0	0
June	1	0	1 (100)	13	1 (8)	5 (38)	58	21 (36)	19 (33)	3	0	1 (33)
July	0	0	0	82	22 (27)	19 (23)	34	14 (41)	7 (21)	1	0	1 (100)
Aug.	23	3 (13)	7 (30)	38	4 (11)	7 (18)	4	0	1 (25)	0	0	0
Sept.	85	22 (26)	13 (15)	1	0	0	1	0	1 (100)	1	0	0
Oct.	6	2 (33)	3 (50)	0	0	0	1	1 (100)	1 (100)	3	0	2 (66)
Total	115	27 (23)	24 (20)	137	27 (19)	31 (23)	110	39 (35)	33 (30)	8	0 (0)	4 (50)

References:

1. Tamura, K. Estimation of the number of nucleotide substitutions when there are strong transition-transversion and G+C-content biases. *Mol. Biol. Evol.* **9,** 678–687 (1992).

2. Kumar, S., Stecher, G. & Tamura, K. MEGA7: Molecular Evolutionary Genetics Analysis version 7.0 for bigger datasets. *Mol. Biol. Evol.* msw054 (2016). doi:10.1093/molbev/msw054

3. Capilla-Lasheras, P. *et al.* Elevated Immune Gene Expression Is Associated with Poor Reproductive Success of Urban Blue Tits. *Front. Ecol. Evol.* **5,** 1–13 (2017).
